# DNA Hypomethylation May Contribute to Metabolic Recovery of Frozen Wood Frog Brains

**DOI:** 10.3390/epigenomes6030017

**Published:** 2022-07-12

**Authors:** Tighe Bloskie, Kenneth B. Storey

**Affiliations:** Department of Biology, Carleton University, Ottawa, ON K1S 5B6, Canada; tighebloskie@cmail.carleton.ca

**Keywords:** DNA methylation, epigenetics, freeze tolerance, metabolic rate depression, *Rana sylvatica*

## Abstract

Transcriptional suppression is characteristic of extreme stress responses, speculated to preserve energetic resources in the maintenance of hypometabolism. In recent years, epigenetic regulation has become heavily implicated in stress adaptation of many animals, including supporting freeze tolerance of the wood frog (*Rana sylvatica*). However, nervous tissues are frequently lacking in these multi-tissue analyses which warrants investigation. The present study examines the role of DNA methylation, a core epigenetic mechanism, in the response of wood frog brains to freezing. We use immunoblot analysis to track the relative expression of DNA methyltransferases (DNMT), methyl-CpG-binding domain (MBD) proteins and ten-eleven-translocation (TET) demethylases across the freeze-thaw cycle in *R. sylvatica* brain, including selected comparisons to freeze-associated sub-stresses (anoxia and dehydration). Global methyltransferase activities and 5-hmC content were also assessed. The data show coordinated evidence for DNA hypomethylation in wood frog brains during freeze-recovery through the combined roles of depressed DNMT3A/3L expression driving lowered DNMT activity and increased TET2/3 levels leading to elevated 5-hmC genomic content (*p* < 0.05). Raised levels of DNMT1 during high dehydration were also noteworthy. The above suggest that alleviation of transcriptionally repressive 5-mC DNA methylation is a necessary component of the wood frog freeze-thaw cycle, potentially facilitating the resumption of a normoxic transcriptional state as frogs thaw and resume normal metabolic activities.

## 1. Introduction

In recent years, crucial roles for epigenetic regulation in supporting hypometabolic states have begun to be established, particularly via DNA methylation and histone modification mechanisms. Evidence for epigenetic control of metabolic rate depression (MRD) has been found in freeze-tolerant anurans [1,2], anoxia-tolerant turtles [3,4], hibernating ground squirrels [5,6,7,8], and selected invertebrates [9]. Given that epigenetics is a major transcriptional control mechanism, and that gene transcription alone utilizes 1–10% of the cell’s total energy budget [10], it is expected that suppressed production of new transcripts is a key part of entrance/maintenance of hypometabolism [11,12] and is mediated by tight epigenetic regulatory mechanisms.

**Figure 1:**
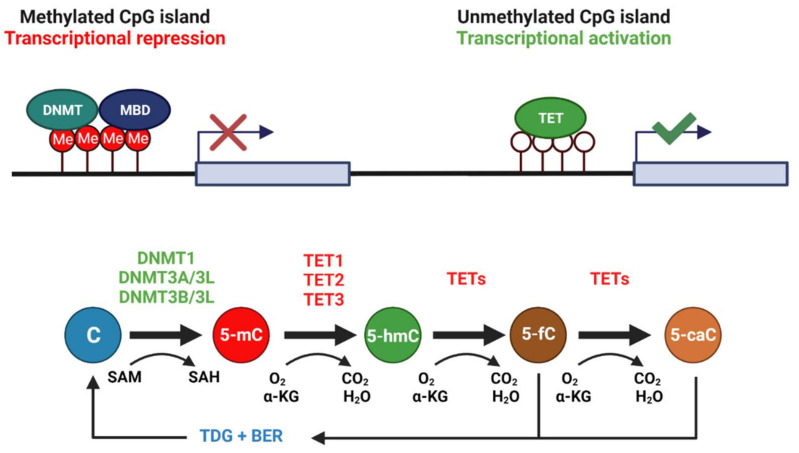
CpG islands exist ubiquitously throughout the genome, often upstream of gene promoter elements. Methylation of CpG islands is typically associated with repression of downstream gene transcription. Cytosine residues in these CpG islands are often methylated by DNMT3A/3L and DNMT3B/3L complexes, whereas DNMT1 functions mainly to maintain 5-mC of hemi-methylated DNA during replication. DNMTs require the universal methyl donor SAM. DNA demethylation requires the coordinated action of TET enzymes with TDG and the base excision repair pathway, generating 5-hydroxymethylcytosine, (5-hmC), 5-fluorocytosine (5-fC), and 5-carboxylcytosine (5-caC) intermediates during the process. TETs require oxygen and α-ketoglutarate (αKG) as reaction substrates. Created with Biorender.com.

DNA methylation is a reversible, core epigenetic mechanism involving the addition/removal of 5-methylcytosine (5-mC) throughout the genome—often at CpG dinucleotides within CpG islands upstream of the promoters of most genes. Hypermethylation of CpG islands correlates with transcriptional silencing of downstream genes, often through the recruitment of repressive methyl-CpG-binding proteins such as MBD1, MBD2, and MeCP2 [13,14]. MBD binding can recruit chromatin remodeling complexes to block the access of transcriptional machinery to promoter elements [15]. Methylation is carried out by DNA methyltransferases (DNMTs) that transfer methyl groups donated from S-adenosyl methionine (SAM). Two major classes of DNMTs exist: (1) maintenance methyltransferases, such as DNMT1, that bind hemi-methylated DNA to ensure that methylation patterns are passed to daughter cells during DNA replication and (2) de novo methyltransferases, such as DNMT3A and DNMT3B, that target unmethylated CpG islands in response to external or internal stimuli of various kinds [16]. However, evidence for DNMT3A and DNMT3B action in maintenance methylation [17,18] and de novo DNMT1 activity at certain loci [19] suggest that these definitions are incomplete. A catalytically inactive third member of the DNMT3 family, called DNMT3L, forms complexes with DNMT3A and DNMT3B to help regulate their activity [20,21]. DNA methylation is dynamic, with 5-mC removal occurring either (1) passively through replication of genetic material or (2) actively through the action of ten-eleven-translocation enzymes (TET1-3) in the presence of O_2_ and α-ketoglutarate [22]. 5-hydroxylmethylcytosine (5-hmC) is the primary stable intermediate of 5-mC in DNA, with active demethylation then completed by the base excision repair (BER) pathway, where thymine DNA glycosylase (TDG) excises later intermediates 5-fluorocytosine (5-fC) or 5-carboxylcytosine (5-caC), resulting in an unmodified cytosine residue [23] (see Figure 1). Indeed, active DNA demethylation provides a site-specific mechanism to regulate transcription in response to environmental stressors, such as freezing, anoxia and dehydration, among others. Given the oxygen-sensitive nature of this DNA modification, in that TET enzymes require O_2_ substrates, it could be a likely regulator involved in *Rana sylvatica* freeze tolerance.

Animal freeze tolerance involves complex responses to several stresses. Freezing halts breathing, heartbeat, blood flow, and muscle movement, causes major water loss from cells into growing extracellular ice masses, and isolates cells/organs from receiving nutrients and oxygen. Hence, at the cell level, freezing blocks delivery of oxygen and nutrients, causes a major reduction in cell volume as water flows out of cells to join extracellular ice masses, and effectively isolates each cell for the duration of the freeze [24]. Ice formation in extracellular spaces leads to passive movement of water out of cells causing cell shrinkage, but a critical minimum cell volume can be sustained by an influx or synthesis of large amounts of colligative cryoprotectants. In the case of wood frogs, the cryoprotectant is glucose and the use of this sugar will also impose hyperglycemia stress on cells. While frozen, cells turn to less efficient anaerobic glycolysis for ATP production but coordinated metabolic rate depression (MRD) lowers energy demands to prevent depletion of limited fuel resources over the course of prolonged freezing. Growing research has outlined numerous mechanisms that contribute to the maintenance of MRD (largely focused on liver or skeletal muscle tissues), ranging from reversible post-translational protein modifications [25,26,27,28] to post-transcriptional miRNA controls [29,30] and increasingly to transcriptional controls via epigenetic mechanisms such as DNA methylation [1]. However, research on the brain (a crucial organ needed to orchestrate thawed recovery of frogs) and into the role of DNA demethylation is currently lacking.

The present study investigates the expression and global activity of DNMT enzymes (DNMT1, DNMT3A, and DNMT3L), as well as the abundance of repressive MBD effectors (MBD1, MBD2, and MeCP2) across the freeze-thaw cycle and associated sub-stresses in wood frog nervous tissue. To appreciate the dynamic nature of epigenetic marks, the study also investigates the expression of active demethylation enzymes, TET2, TET3, and TDG, across the freeze-thaw cycle. The abundance of genomic 5-hmC is also compared across the freeze-thaw cycle to determine whether enzyme expression translates into functional changes on a genome-wide scale. The results give strong indications of robust gene activation during recovery from freezing, with coordinated roles of depressed DNA methylation and induced demethylation.

## 2. Results

### 2.1. DNMT Expression Consistent with the Transcriptional State of Cells across the Wood Frog Freeze-Thaw Cycle

**Figure 2:**
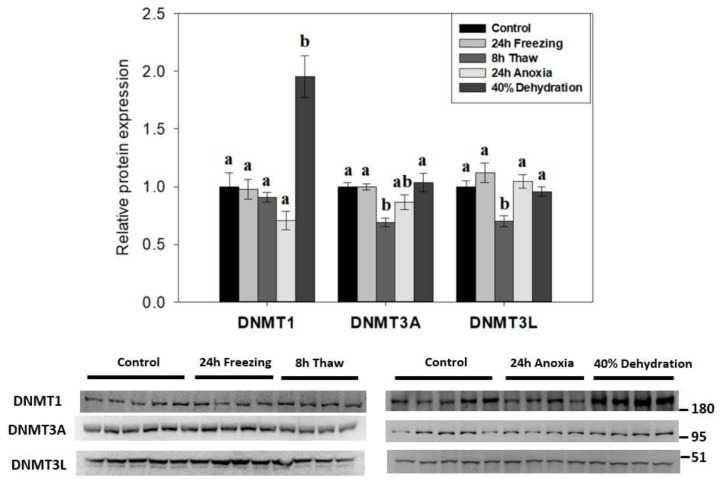
Effects of 24 h freezing, 8 h thaw, 24 h anoxia, and 40% dehydration on total protein levels of DNA methyltransferases DNMT1, DNMT3A, and DNMT3L in brain tissue of *Rana sylvatica* as determined by Western immunoblotting. Representative immunoblots are also shown, included with the approximate location of the closest molecular marker. DNMT3B antibodies did not cross-react strongly with protein homogenates in the target range. Data are mean ± SEM, on *n* = 4–5 independent trials on tissue samples from different animals. Values labeled with different letters (a, b) are significantly different from one another (*p* < 0.05). DNMT3A is *n* = 5 for frozen samples, while other targets are *n* = 4.

To determine if DNA methylation played a role in the response of wood frog nervous tissue in animals exposed to whole body freezing, the relative expression of canonical DNA methyltransferase family members (DNMT1, DNMT3A, DNMT3L) was examined across the freeze-thaw cycle and associated sub-stresses in *R. sylvatica* brain total protein homogenates using immunoblotting (Figure 2). De novo methyltransferase partners DNMT3A/3L showed similar patterns with reductions in expression during post-freeze thawing (to 69 ± 4% and 70 ± 5%, respectively; *p* < 0.05) as compared to controls. However, 8-h thawed DNMT3A levels were not significantly different from levels in wood frog brains exposed to anoxia. Interestingly, DNMT1 levels in brains of frogs dehydrated of 40% of total body water were greatly elevated in comparison to all other experimental conditions (up 1.95 ± 0.18-fold compared with controls; *p* < 0.05) and DNMT1 was the only one of the three DNMTs that responded to dehydration stress.

### 2.2. Noticeable Depression in DNA Methyltransferase Activity during Freezing, Freeze Recovery, and Anoxia

**Figure 3:**
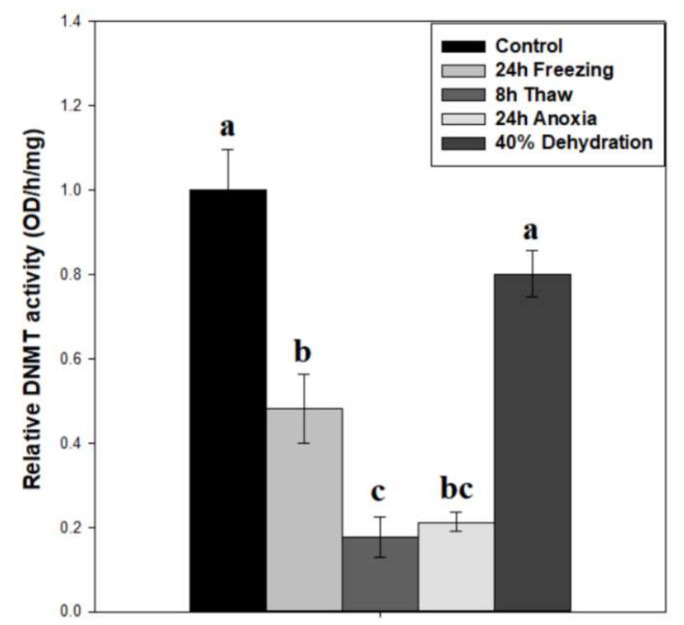
Relative DNMT total enzymatic activity in nuclear extracts of brain tissue from control, 24 h frozen, 8 h thawed, 24 h anoxic, and 40% dehydrated *Rana sylvatica*. Relative enzyme activities are shown. Data are mean ± SEM, *n* = 5 independent trials on samples from different animals. Actual quantified control activity was 5.19 ± 0.47 OD units/h/mg. Values labeled with different letters (a–c) are significantly different from one another (*p* < 0.05).

To test whether DNMT activity correlated with protein expression, total DNMT activity was measured on wood frog brain nuclear extracts across the freeze-thaw cycle and associated sub-stresses (Figure 3). Our results indicate a decreasing pattern of activity across the freeze-thaw cycle, with relative DNMT activity levels down to 64 ± 7% after 24-h freezing (*p* < 0.05), and a further reduction to 31 ± 5% during freeze recovery (*p* < 0.05), relative to controls. The nuclear samples from 24-h anoxia exposure also showed very low DNMT activities that were not significantly different from the 24 h frozen or 8 h thawed conditions, whereas DNMT activities in samples from brain of dehydrated frogs were unchanged as compared with controls. The difference in DNMT activity between 24 h frozen and 24 h anoxic nuclear samples was 2.26-fold (*p* = 0.062).

### 2.3. Methyl-CpG Binding Protein Expression Unchanged across the Freeze-Thaw Cycle

**Figure 4:**
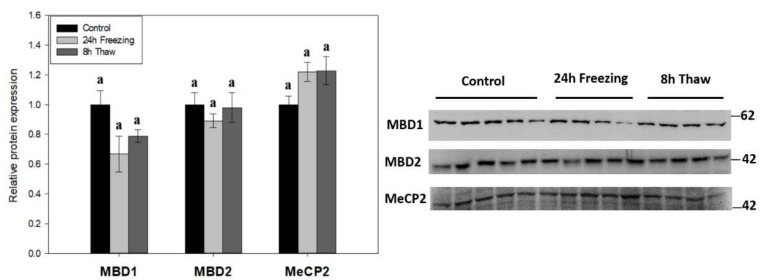
Relative expression of methyl-CpG-binding proteins MBD1, MBD2, and MeCP2 across the freeze-thaw cycle in brain tissue of *Rana sylvatica* as determined by Western immunoblotting. Representative immunoblot bands are also shown, included with the approximate location of the closest molecular marker. Data are mean ± SEM, for *n* = 4–5 independent trials on tissue samples of different animals. Statistical testing revealed no significant differences between the experimental conditions for any of the three proteins. MBD2 and MeCP2 are *n* = 5 for frozen samples, while MBD1 is *n* = 4.

The expression of methyl-CpG-binding proteins MBD1, MBD2, and MeCP2 was tested across the freeze-thaw cycle in wood frog brain tissue by immunoblotting to link DNA methylation activity with “reader” proteins (Figure 4). The levels of all three effector proteins were unchanged during freezing and thawed recovery.

### 2.4. Multi-Faceted Support for Increased DNA Demethylation during Freeze Recovery

**Figure 5:**
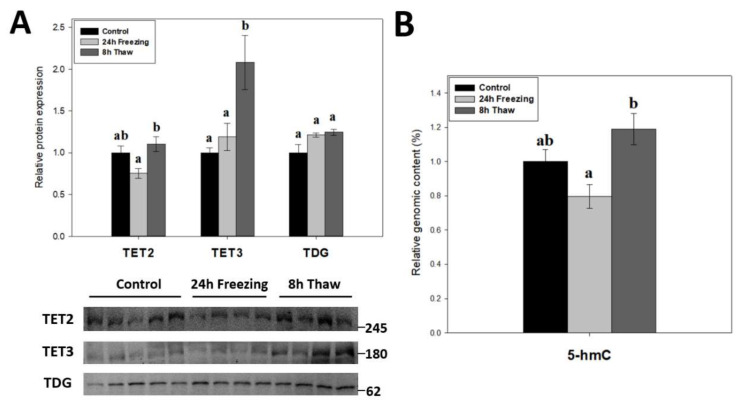
(**A**) Relative expression of DNA demethylation enzymes TET2, TET3, and TDG across the freeze-thaw cycle in brain tissue of *Rana sylvatica* as determined by Western immunoblotting. Representative immunoblot bands are also shown, included with the approximate location of the closest molecular marker. Data are mean ± SEM, *n* = 4–5 independent trials on tissue samples of different animals. TET1 antibodies did not cross-react strongly with protein homogenates in the target range. (**B**) Relative genomic 5-hmC content in brain tissues from control, 24 h frozen, and 8 h thawed *Rana sylvatica*. Data are mean ± SEM, *n* = 5 independent trials on 15 ng of DNA samples from different animals. Values labeled with different letters (a, b) are significantly different from one another (*p* < 0.05).

Demethylation factors were also assessed across the brain freeze-thaw cycle given the dynamic nature of DNA methylation. The relative protein levels of TET2, TET3, and TDG were measured over the freeze-thaw cycle using immunoblotting (Figure 5A). The results indicate dual up-regulation of TET2 and TET3 expression during thawed recovery (*p* < 0.05); values increased by 1.47-fold and 1.75-fold versus comparable frozen levels, respectively. Thawed TET3 levels were also significant (*p* < 0.05) from controls, while TET2 was not. However, TDG levels were not affected by either freezing or thawing. To test whether the changes in TET expression were linked to DNA demethylation, the relative 5-hmC content of genomic DNA was also measured across the freeze-thaw cycle of wood frog brain tissue (Figure 5B). In agreement with the results for TETs, the relative 5-hmC content rose significantly for gDNA from thawed versus frozen frogs (*p* < 0.05), providing good evidence for enhanced DNA demethylation during freeze recovery.

## 3. Discussion

Recent studies on freeze-tolerant and anoxia-tolerant animals have identified epigenetic mechanisms as potential regulators of global transcriptional repression [1,2,3,4], a hallmark of metabolic rate depression [5,11,12]. Similarly, epigenetic controls have been linked to stress recovery states, with evidence for rebounded transcriptional activation. Methylation of histone lysine/arginine residues, of DNA cytosines and of mRNA adenosines highlight the current research, with notable gaps in demethylation pathways. Additionally, nervous tissue is largely understudied in the field at this time. The present work addresses both needs by investigating the role of reversible DNA methylation in the freeze-tolerance of wood frog brain. Specifically, this work measured the expression of DNMT1/3 enzymes along with global methyltransferase activity. DNA methylation mechanisms were further explored through analysis of the relative levels of key methyl-CpG-binding proteins MBD1, MBD2, and MeCP2. The expression of TET2/3 demethylases, TDG, which acts in base excision repair, and the consequential 5-hmC genomic content was also examined to inspect the reversible nature of the epigenetic mark. This research builds on preliminary DNA methyltransferase research on liver and skeletal muscle of wood frogs during hypometabolism [1].

### 3.1. Diverse Roles for DNA Methyltransferases in the Brain during Wood Frog Freeze Tolerance

The relative expression of three canonical DNA methyltransferase enzymes was tracked across the freeze-thaw cycle in wood frog brain tissue, along with related responses to anoxia and dehydration sub-stresses (Figure 2). The results show decreases in DNMT3A and DNMT3L expression after 8 h thawed recovery (*p* < 0.05). Interestingly, a strong up-regulation of maintenance DNMT1 expression occurred in response to dehydration stress. However, none of the three DNMT proteins responded significantly to freezing or anoxia stresses in wood frog brain. In linking protein expression with total DNA methyltransferase activity, these parameters were somewhat at odds in wood frog brain under stress conditions. Enzyme activity was strongly suppressed (Figure 3), whereas protein levels were largely unaffected (Figure 2). This conflict could be a result of post-translational modification of one or more of the enzymes, leading to reduced activity during freezing, thawing, or anoxia conditions. Our data also point to anoxia, and not dehydration, as the underlying sub-stress contributing to repressed DNMT activity in frozen wood frog brains.

During the 8-h thaw, the relative expression of DNMT3A and DNMT3L, which form a catalytic hetero-tetramer complex [31], were both reduced relative to control values (Figure 2). It is likely that these reduced DNMT3A/3L levels are a crucial factor in suppressed methyltransferase activity during freeze recovery (Figure 3). DNMT3L stimulates DNMT3A de novo activity of CpG dinucleotide methylation, with a characteristic repressive role on transcription [21]. Reductions in DNMT3A, DNMT3L, and DNMT activity during recovery from freezing is not surprising, and may facilitate the return to standard transcriptional activity under normal aerobic metabolism. It may also activate the expression of increased defense and repair mechanisms following freezing damage.

DNMT1 expression was sharply increased during dehydration stress (Figure 2), a result that was somewhat unexpected. DNMT1 acts primarily in maintenance methylation during DNA replication, with an increased affinity towards hemi-methylated CpG substrates [32]. In the non-proliferative state of wood frog brain under high dehydration, the role of increased expression of this maintenance methyltransferase is difficult to explain. However, de novo activities for DNMT1 have been shown more recently [19,33], particularly in response to vertebrate oxidative stress [34]. Interestingly, DNMT1 levels were unchanged in 24-h freezing and anoxia stresses (Figure 2) that also pose oxidative stressors. The difference may lie in stress response timelines: responses to freezing and anoxia are rapid (minutes to a few hours), whereas dehydration is a slow, gradual loss of body fluids over several days. We propose elevated DNMT1 may contribute to transcriptional silencing in prolonged, long-term hypometabolism. The present results also suggest that increased DNMT1 expression may partly answer why global DNA methyltransferase activity is not depressed during dehydration but is strongly reduced under freezing and anoxia stresses (Figure 3).

Considering that none of the investigated DNMT enzymes showed reduced protein levels under stress conditions, despite suppressed DNMT activity in both frozen and anoxic brain, it can be proposed that post-translational modifications of DNMTs are driving regulation of these proteins under environmental stress conditions. For example, DNMT1 is negatively regulated by protein kinase C (PKC)-mediated phosphorylation of N-terminal serine residues [35], and PKC may be hyperactive in wood frog brain during freezing [36]. Similarly, DNMT3A is phosphorylated by casein kinase 2 (CK2) at S386 and S389 to repress its activity [37]. CK2 is an under-investigated factor in freeze-tolerance at the present time, but its implication in cell cycle control, DNA repair, and circadian rhythm regulation [38] make it particularly noteworthy for future study. Reduced DNA methyltransferase activity was similarly found in liver of wood frogs under hypometabolic conditions [1].

### 3.2. Thaw-Mediated Hyperactive DNA Demethylation Relative to Freezing

To investigate the role of active DNA demethylation in adaptive strategies, the expression of TET2/3 and TDG (Figure 5A) as well as the relative 5-hmC genomic content (Figure 5B) were tracked across the freeze-thaw cycle of wood frog brain tissue. The transition from freezing to thawing drove significant increases in TET2, TET3, and relative 5-hmC levels (*p* < 0.05) as compared with values in the frozen state, which is consistent with the proposed transcriptional status under both conditions [39]. It should be noted, however, that neither frozen nor thawed TET2 and 5-hmC levels were significantly different from controls. The above results suggest that enhanced DNA demethylation action helps to alleviate repressive CpG methylation and may facilitate a return to active transcription during thawed recovery. This correlates with earlier results showing lowered DNMT3A and DNMT3L protein levels and reduced DNMT activity during recovery after freezing.

TET enzymes require O_2_ as a substrate in subsequent oxidations of the 5′-methyl group on cytosine residues. Given their oxygen-sensitive nature, elevated 5-hmC levels (relative to freezing) (Figure 5B) during reperfusion is not surprising. Interestingly, oxygen-dependent 5-hydroxymethylation of the TET3 promoter in embryonic stem cells actually induces its own expression [40], which might partially explain the strong induction of TET3 levels during 8-h thawing. Moreover, HIF-1α knockdown, which is observed in freeze recovery of another freeze tolerant animal *Eurosta solidaginis* [41], has been shown to induce TET2 expression and demethylase activity [42]. TET enzymes have also been linked to the DNA damage response; particularly in double-strand break repair [43,44]. Elevated ROS production during reoxygenation, which has been shown to cause raised DNA damage in wood frog livers [45], likely requires the induction of DNA damage responses, in which increased TET expression may be involved. Additionally, TET3 is implicated in cell cycle control by promoting cyclin D1 expression [46]. Indeed, cyclin D1 expression is strongly induced during freeze recovery [47], which may be under TET3 control.

Induction of TET2/3 protein expression during freeze recovery (relative to freezing) may be due to alleviation of post-transcriptional controls. In particular, TET2 is regulated at the post-transcriptional level by microRNAs miR-22, miR-29, miR-101, and miR-125 [48,49]. Indeed, miRNA-seq analysis of wood frog brains showed that relative expression of miR-22 and miR-125 were reduced during thawing after a 24 h freeze [50]. Therefore, it can be hypothesized that TET2 induction during recovery from freezing may be due, at least in part, to the mitigation of repressive miR-22 and miR-125 silencing controls.

## 4. Materials and Methods

### 4.1. Animal Experiments

Adult male wood frogs (*Rana sylvatica*), of 5–7 g mass, were captured near Ottawa, Ontario at breeding ponds during early spring. Frogs were washed in tetracycline and held in plastic boxes with damp sphagnum moss, and acclimated (unfed) for two weeks at 5 °C. Control frogs were sampled from this group. For freezing treatment, sub-sets of remaining frogs were placed into plastic boxes with a layer of damp paper towel on the bottom, and transferred to an incubator set to −4.0 °C for 45 min to allow ice nucleation on the skin due to contact with ice crystals forming in the paper. Incubation temperature was subsequently raised to −2.5 °C for 24 h. This treatment results in freezing of 65–70% of total body water as extracellular ice [24]. Half of the frogs were then sampled while frozen, whereas the remaining frogs were returned to a 5 °C incubator for 8 h and allowed to fully thaw before sampling.

Anoxia stress was as previously described [51]. Separately, damp paper towels from distilled water bubbled with 100% nitrogen gas for roughly 30 min lined the bottom of incubation chambers. Chambers were flushed with 100% N_2_, then 5 °C acclimated frogs were added to anoxic containers sealed with parafilm and left for 24 h. Anoxic frogs were sampled.

Dehydration stress was as previously described [52]. Animals were weighed and placed into dry buckets (without lids) at 5 °C. Frogs lost body water via evaporation across the skin and were weighed at regular 8–12 h intervals. Animals were sampled when ~40% of total body water was lost. The percentage of body water lost was calculated by the following formula:

$$\%Dehydration=\frac{M_{i}-M_{d}}{M_{i}-BWC_{i}}\times 100\%$$

where *M_i_* is the initial frog body mass, *M_d_* is the dehydrated frog mass, and *BWC_i_* is the initial body water content (0.808 ± 0.012 g H_2_O per g body mass).

Frogs sampled from all three treatment groups were euthanized by pithing, and brain tissue was quickly extracted and flash-frozen in liquid N_2_. Tissues were subsequently stored at −80 °C until use. All protocols were approved by the Carleton University Animal Care Committee (protocol #106935) and followed the guidelines set by the Canadian Council on Animal Care.

### 4.2. Total Soluble Protein Isolation

Total soluble protein was extracted from control, 24 h frozen, 8 h thawed, 24 h anoxic, and 40% dehydrated frogs. Frozen samples of brain (*n* = 5 biological replicates) of near equal weight (~80 mg) were homogenized (1:5 *w*/*v*) in chilled 1× lysis buffer (EMD Millipore, Cat# 43–045, Billerica, MA, USA) using a Polytron PT10. Lysis buffer was supplemented with inhibitors; 1 mM Na_3_VO_4_, 10 mM NaF, 10 mM β-glycerophosphate, and 10 μL/mL protease inhibitor cocktail (Bioshop, Cat# PIC002, Burlington, ON, Canada). Protein homogenates were centrifuged at 14,000× *g* for 20 min at 4 °C, and supernatants were collected.

Protein was quantified using the BioRad protein assay (BioRad Laboratories, Cat# 5000002, Hercules, CA, USA). Concentrations were standardized to 10 μg/μL with 1× lysis buffer. Samples were then mixed 1:1 *v/v* with 2× SDS buffer (100 mM Tris-base, 4% *w/v* SDS, 20% *v/v* glycerol, 0.2% *w/v* bromophenol blue, 10% *v/v* 2-mercaptoethanol), denatured by boiling for 10 min, and immediately placed on ice. A small aliquot was removed to verify protein integrity via SDS-PAGE, whereas the rest of the samples were stored at −40 °C until use.

### 4.3. Nuclear Protein Extraction

Roughly 80 mg samples of frozen *R. sylvatica* pooled brain tissue (*n* = 5 independent replicates per experimental condition) were crushed in liquid N_2_, and manually homogenized 1:4 *w/v* in chilled cytosolic fraction buffer (10 mM HEPES, 10 mM KCl, 10 mM EDTA, 20 mM β-glycerophosphate, 1 mM DTT, 0.1% Triton X-100, and 10 µL/mL protease inhibitor cocktail (Bioshop, Cat# PIC002, Burlington, ON, Canada), pH 7.9) using a Dounce homogenizer. Homogenates were centrifuged at 10,000× *g* for 15 min at 4 °C, and then cytoplasmic supernatants were collected. Pellets were washed with cytosolic buffer then sonicated in 250 µL of nuclear fraction buffer (20 mM HEPES, 400 mM NaCl, 1 mM EDTA, 10% *v/v* glycerol, 20 mM β-glycerophosphate, pH 7.9). Samples were spun at 10,000× *g* for 15 min at 4 °C, and then nuclear supernatants were transferred to fresh tubes. Aliquots of nuclear and cytoplasmic protein levels were removed to quantify protein levels using the BioRad protein assay (BioRad Laboratories, Cat# 5000002, Hercules, CA, USA). Remaining samples were then stored at −70 °C until use in enzymatic assays. Selected samples were also tested via immunoblotting to assess cytoplasmic (α-tubulin) and nuclear (histone H3) markers in order to validate an effective separation of nuclear and cytosolic fractions.

### 4.4. Immunoblotting

Samples of equal protein amount (10–50 μg; optimized for each target) were loaded onto discontinuous SDS-PAGE gels with 4–7 μL of PiNK Plus pre-stained protein ladder (10.5–175 kDa; FroggaBio, Cat# PM005-0500K, Concord, ON, Canada) or BLUeye pre-stained protein ladder (10–245 kDa; FroggaBio, Cat# PM007-0500K, Concord, ON, Canada) as molecular weight markers. Stacking gels were 5% acrylamide *v/v* in 1 M Tris buffer (pH 6.8) with 0.1% SDS, 0.1% APS and 0.1% TEMED. Separating gels contained 8–15% acrylamide *v/v* in 1.5 M Tris buffer (pH 8.8), with 0.1% SDS, 0.1% APS, and 0.1% TEMED. Proteins were separated using the BioRad Mini Protean III system (BioRad Laboratories, Hercules, CA, USA) in running buffer (25 mM Tris-base, 190 mM glycine, 0.1% *w/v* SDS, pH 8.5) at 180 V for 45–150 min. Resolved protein was electroblotted onto 0.45 μm polyvinylidene difluoride membranes by wet transfer at 160 mA for 45–150 min in transfer buffer (25 mM Tris-base, 190 mM glycine, 10% *v/v* methanol, pH 8.5).

Membranes were blocked for 30 min in 1–5% skim milk *w/v* in 1× TBST (20 mM Tris-base, 140 mM NaCl, 0.05% Tween-20) to discourage non-specific antibody binding, followed by 3 × 5 min washes in 1× TBST. Membranes were then probed overnight on a rocker with primary antibodies (diluted 1:1000 to 1:5000 *v/v* as needed) in TBST with 0.02% sodium azide at 4 °C. Excess antibody was removed by 3 × 5 min washes in 1× TBST and membranes then probed by 1:5000 or 1:8000 *v/v* with HRP-linked goat anti-rabbit secondary antibodies (BioShop, Cat# APA002P, Burlington, ON, Canada) in TBST at room temperature for 30 min. Following final 3 × 5 min washes in 1× TBST, membranes were visualized by chemiluminescence (H_2_O_2_ and Luminol) using the ChemiGenius Bio Imaging System (Syngene, Frederick, MD, USA). Band intensities were standardized against representative total protein levels in each well after staining with Coomassie (0.25% *w/v* Coomassie brilliant blue, 7.5% *v/v* acetic acid, 50% methanol) as a loading control. The expression of key DNA methyltransferases (DNMT1, DNMT3A, and DNMT3L), methyl-CpG-binding domain proteins (MBD1, MBD2, and MeCP2), and demethylation enzymes (TET2, TET3, and TDG) were measured across the freeze-thaw cycle, and selectively for anoxic and dehydrated sub-stresses. See Table 1 for the full list of antibodies used in immunoblotting.

### 4.5. Quantification and Statistics

Chemiluminescent bands were quantified by densitometry using a ChemiGenius Bio Imaging System and GeneTools Software (version 4.3.8.0, Syngene, Frederick, MD, USA). Band intensities in each lane were standardized against a large group of Coomassie-stained bands well-separated from the bands of interest. Data are expressed as mean ± SEM, *n* = 4–5 samples from pooled brains of different animals. Statistical analysis was performed by one-way ANOVA and a Tukey’s post-hoc test (*p* < 0.05) using SigmaPlot 12.0 statistical software (Systat Software Inc., San Jose, CA, USA).

### 4.6. DNMT Activity ELISA

DNMT activity was quantified using the EpiQuik DNMT Activity/Inhibition Assay Ultra Kit (Epigentek, Cat# P-3009, Farmingdale, NY, USA), following manufacturer guidelines. All necessary reagents were supplied with the ELISA kits. A dilution series of pooled nuclear extracts (10–40 µg) was used to optimize ideal absorbance readings for the kits. Blank and control enzyme (300 µg/mL; Epigentek) wells were also run. For samples, 30 µg each of nuclear extracts from *n* = 5 control, 24 h frozen, 8 h thawed, 24 h anoxic, and 40% dehydrated conditions were added to wells supplied with DNMT substrates and incubated for 2 h at 37 °C. Wells were then washed three times in 1× wash buffer, before 5-mC capture antibody was added to each well and incubated for 1 h at room temperature. The wells were again washed three times and incubated in detection antibody for 30 min at room temperature. Following four washes in 1× wash buffer, the enhancer solution was incubated in target wells for 30 min at room temperature. After a final five washes, the supplied developer solution was incubated in each well until positive control wells turned deep blue, at which point the supplied Stop Solution was added to each well and absorbance was read at 450 nm. The total DNMT activity was calculated using the following formula:

$$DNMT\;Activity\;\left(OD/h/mg\right)=\frac{Sample\;A450-Blank\;A450}{Protein\;Amount\;\left(ug\right)\times Incubation\;Time\;\left(h\right)}\times 1000$$

### 4.7. Genomic DNA Extraction

Genomic DNA was extracted from 25 mg samples of frozen tissue (*n* = 5 per experimental condition) using the Quick-DNA Miniprep Plus Kit (Zymo Research, Cat# D4068, Irvine, CA, USA) according to manufacturer directions for tissue samples. Briefly, tissues were digested with proteinase K overnight at 55 °C, then centrifuged at 12,000× *g* for 1 min, and the aqueous supernatant containing DNA was transferred and bound to the supplied spin column. Spin columns were washed multiple times with the provided wash buffer, then eluted in heated DNA elution buffer (10 mM Tris-HCl, 0.1 mM EDTA, pH 8.5). Genomic DNA samples were measured using a Take3 Micro-Volume plate (BioTek, Winooski, VT, USA). All samples had a 260/280 ratio >1.8, with concentrations ranging from 20–70 ng/µL, and DNA integrity was validated on a 0.5% agarose gel.

### 4.8. 5-Hydroxymethylcytosine ELISA

Genomic 5-hmC content was quantified using the Global 5-hmC DNA ELISA kit (Active Motif, Cat# 55025, Carlsbad, CA, USA), as per manufacturer guidelines. All necessary reagents were included with ELISA kits. Briefly, genomic DNA (*n* = 5 per experimental condition) was fragmented via *MseI* digestion at 37 °C for 3 h. To coat the wells, supplied DNA binding agent was added to wells and incubated for 1 h at room temperature. A 15 ng aliquot of denatured genomic DNA was loaded per well, this amount being previously shown to be within the linear range of a pooled sample dilution series. A standard curve was also run via serial dilutions of a 5-hmC standard. Wells containing gDNA, as well as blank and standard curve wells, were incubated at 37 °C for 2 h to enable gDNA binding. With three washes between each subsequent step, wells were incubated with 5-hmC primary antibody (1:2000 *v*/*v*) and anti-rabbit HRP-conjugated secondary antibody (1:4000 *v*/*v*) for 1 h at room temperature with mild agitation. Finally, wells were incubated with developing solution until a range of darkening blue could be seen within the standard curve wells, at which point the stop solution was added. OD450 and the reference wavelength OD655 were measured. The percentage of 5-hmC was calculated by the following equations:

$$ng\;5–hmc=\frac{Norm.\;\;Sample\;OD-Y-intercept}{Slope\times 9.5}\;\%\;5–hmc=\frac{ng\;5-hmc}{ng\;DNA\;input}\times 100$$

Note: 9.5 is a standardization factor to account for the standard only containing 9.5% 5-hmC.

## 5. Conclusions

In summary, this study is the first to highlight DNA methylation regulatory mechanisms in a nervous tissue (brain) of a vertebrate species that endures multiple environmental stresses (freezing, anoxia, and dehydration) with specific adaptive strategies including metabolic rate depression. The present results provide strong evidence in favor of DNA hypomethylation during recovery after freezing as supported by lower DNMT3A and DNMT3L protein levels, strongly reduced DNMT activities, and increased TET2 and TET3 expression as well as elevated 5-hmC content. Elevated DNMT1 during dehydration was also noteworthy. These mechanisms may contribute to the rebound state of transcription in cells during recovery after freezing.

## Figures and Tables

**Table 1:** Antibody information.

Target	Company	Catalogue #
DNMT1	Abclonal	#A16729
DNMT3A	Genetex	#GTX128157
DNMT3B *	Genetex	#GTX129127
DNMT3L	Abgent	#AP1040a
MBD1	Active Motif	#39858
MBD2	Active Motif	#39548
MeCP2	Active Motif	#39189
TET1 *	Genetex	#GTX124207
TET2	Abclonal	#A5682
TET3	Genetex	#GTX121453
TDG	Genetex	#GTX110473

* Both DNMT3B and TET1 were tested in this study, but neither antibody showed strong cross-reactivity with protein homogenates in the target range.

## Data Availability

The data that support the findings of this study are available from the corresponding author upon reasonable request.
